# Controllable Preparation of V_2_O_5_/Graphene Nanocomposites as Cathode Materials for Lithium-Ion Batteries

**DOI:** 10.1186/s11671-016-1764-3

**Published:** 2016-12-12

**Authors:** Yanglin Liu, Yaping Wang, Yifang Zhang, Shuquan Liang, Anqiang Pan

**Affiliations:** 1 School of Materials Science and Engineering, Central South University, Changsha, 410083 Hunan China; 2 Changsha Environmental Protection Vocational College, Changsha, 410004 Hunan China

**Keywords:** Vanadium oxides, Reduced graphene oxide, Lithium-ion batteries, Nanosheets, Nanoparticles

## Abstract

Transition metal oxides and graphene composites have been widely reported in energy storage and conversion systems. However, the controllable synthesis of graphene-based nanocomposites with tunable morphologies is far less reported. In this work, we report the fabrication of V_2_O_5_ and reduced graphene oxide composites with nanosheet or nanoparticle-assembled subunits by adjusting the solvothermal solution. As cathode materials for lithium-ion batteries, the nanosheet-assembled V_2_O_5_/graphene composite exhibits better rate capability and long-term cycling stability. The V_2_O_5_/graphene composites can deliver discharge capacities of 133, 131, and 122 mAh g^−1^ at 16 C, 32 C, and 64 C, respectively, in the voltage range of 2.5–4.0 V vs. Li/Li^+^. Moreover, the electrodes can retain 85% of their original capacity at 1C rate after 500 cycles. The superior electrochemical performances are attributed to the porous structures created by the connected V_2_O_5_ nanosheets and the electron conductivity improvement by graphene.

**Electronic supplementary material:**

The online version of this article (doi:10.1186/s11671-016-1764-3) contains supplementary material, which is available to authorized users.

## Background

Rechargeable lithium-ion batteries, as one of the most important energy storage devices, have been widely used in consumer electronic devices such as cell phones, laptop computers, and hybrid electrical vehicles. However, the increasing demands for better batteries push the researchers to develop new electrode materials with improved performance including higher energy density, better rate capability, and longer lifespan [1–3].

Among the numerous cathode materials, V_2_O_5_ is a promising material because of its low cost, abundant resource reservation, and high-energy density [4]. However, the low ionic diffusivity (10^−12^–10^−13^ cm^2^ s^−1^) and moderate electrical conductivity (10^−12^–10^−13^ S cm^−1^) limit its electrochemical performance [5]. To overcome the drawbacks, great efforts have been endeavored to fabricate nanostructured materials. To date, various V_2_O_5_ nanomaterials, such as nanorods [6], nanotubes [7], nanowires [5, 8], nanosheets [9, 10], nanospheres [11–13], and nanoflowers [14, 15] were reported with enhanced electrochemical performance due to the kinetics improvement for redox reactions. However, the intrinsic low electronic conductivity of V_2_O_5_ is still undressed. More recently, making V_2_O_5_ nanocomposites with conductive materials are effective for obtaining high-performance electrode materials. In particular, carbonaceous materials such as carbon nanotubes [16, 17], mesoporous carbon [18], and graphene [8, 19, 20] are of great popularity in making the nanocomposites. Among them, graphene has attracted particular interest in making nanocomposites due to its excellent physical and chemical properties, including high electronic conductivity, high surface area, and superior mechanical properties. The combination between graphene and nanomaterials can not only increase the conductivity and specific surface area of active materials but also prevent them from agglomerating upon cycling [21–23]. In the past years, vanadium oxides and graphene nanocomposites, such as VO_2_@graphene [24], V_2_O_3_@graphene [25], and V_3_O_7_@graphene [26], have been successfully prepared. However, the controllable preparation of vanadium oxides on graphene nanosheets with different structures is rarely reported, which has a big effect on the electrochemical properties.

Herein, we report the structural engineering of V_2_O_5_/graphene with different morphologies by a solvothermal approach with subsequent annealing in air. Vanadium precursors with nanosheet or nanoparticle morphology are controllably prepared on the surface of graphene and can be converted into V_2_O_5_/reduced graphene oxide nanocomposites with good structural reservation in the calcination process in air. As cathode materials for lithium-ion batteries, the nanosheet-structured V_2_O_5_/reduced graphene oxide nanocomposites exhibit much better rate capability and cyclic stability than V_2_O_5_ nanoparticles/reduced graphene oxide composites. The relationship between nanostructures and their electrochemical performance is discussed.

## Methods

### Materials Synthesis

The graphene oxide was made by a modified Hummers method [27]. For the composite synthesis, 10 mg dried graphene oxide (GO) nanosheets were dispersed in 30 mL of isopropyl alcohol by ultra-sonication for 30 min. Then, 0.4 mL of vanadium triisopropoxy oxide (Alfa Aesar) was added into the solution immediately. The mixture was stirred for 10 min before transferring to a 50-mL Teflon-lined stainless steel autoclave, which was later kept at 200 °C for 12 h. After cooling down naturally, the precursor precipitates were collected by centrifugation and washed several times by alcohol, before drying at 60 °C for 12 h. Finally, the dried precursor was annealed at 320 °C for 1 h in air. The obtained material was designated as nanoparticle-structured V_2_O_5_/reduced graphene oxide composite I (V/GO-I).

The nanoparticle-assembled V_2_O_5_/graphene composite was prepared by adding extra 2 mL deionized (DI) water into the solvothermal solution, keeping other parameters unaltered. The obtained material was designated as nanoparticle-structured V_2_O_5_/reduced graphene oxide composite II (V/GO-II).

### Materials Characterization

The crystal phases were collected using a Rigaku D/max2500 with Cu-Kα radiation (*λ* = 1.54178 Å) using a step of 0.02^o^ between 10° and 80° (2θ). The morphologies of the samples were studied by scanning electron microscopy (SEM, FEI Nova Nano SEM 230) and transmission electron microscopy (TEM, FEI Tecnai G20). The X-ray diffraction (XRD) patterns of the samples were collected in the range between 10^o^ and 80^o^ with a step size of 0.02°. The weight percentages of graphene in the V_2_O_5_/graphene composites were determined by thermogravimetric (TG) analysis with a heating rate of 10 °C min^−1^. A spectrometer (Raman, LabRAM HR800) with a back-illuminated charge-coupled detector attachment was used to record the Raman spectra.

### Electrochemical Measurement

The electrochemical performances of the electrodes were tested in a coin cell assembly (2016 type coin cell). For the electrode preparation, a mixture of V_2_O_5_/reduced graphene oxide composites, acetylene black, and polyvinylidene fluoride (PVDF) in a weight ratio of 70:20:10 was dispersed in an *N*-methyl-2-pyrrolidone (NMP) solution to make a slurry, which was coated on aluminum foil and dried in a vacuum oven at 100 °C overnight. The cells were assembled in the glove box (Mbraun, Germany) filled with ultra-high-purity argon using polypropylene membrane as the separator, lithium metal as the anode, and 1-M LiPF_6_ in ethyl carbonate/dimethyl carbonate (1:1 *v*/*v*) as the electrolyte. The cyclic voltammetry (CV) measurement was tested on CHI660C (CH Instrument Electrochemical Workstation), and the galvanostatic discharging/charging experiment was tested on a Land battery tester (Land CT 2001A, Wuhan, China). The measurements were all carried out in the voltage range of 2.5–4.0 V (vs. Li/Li^+^). The electrochemical impedance spectrometry (EIS) was carried out on a ZAHNER-IM6ex electrochemical workstation (ZAHNER Co. Germany) in the frequency range of 100 kHz to 10 mHz using the fresh cell.

## Results and Discussion

**Fig. 1:**
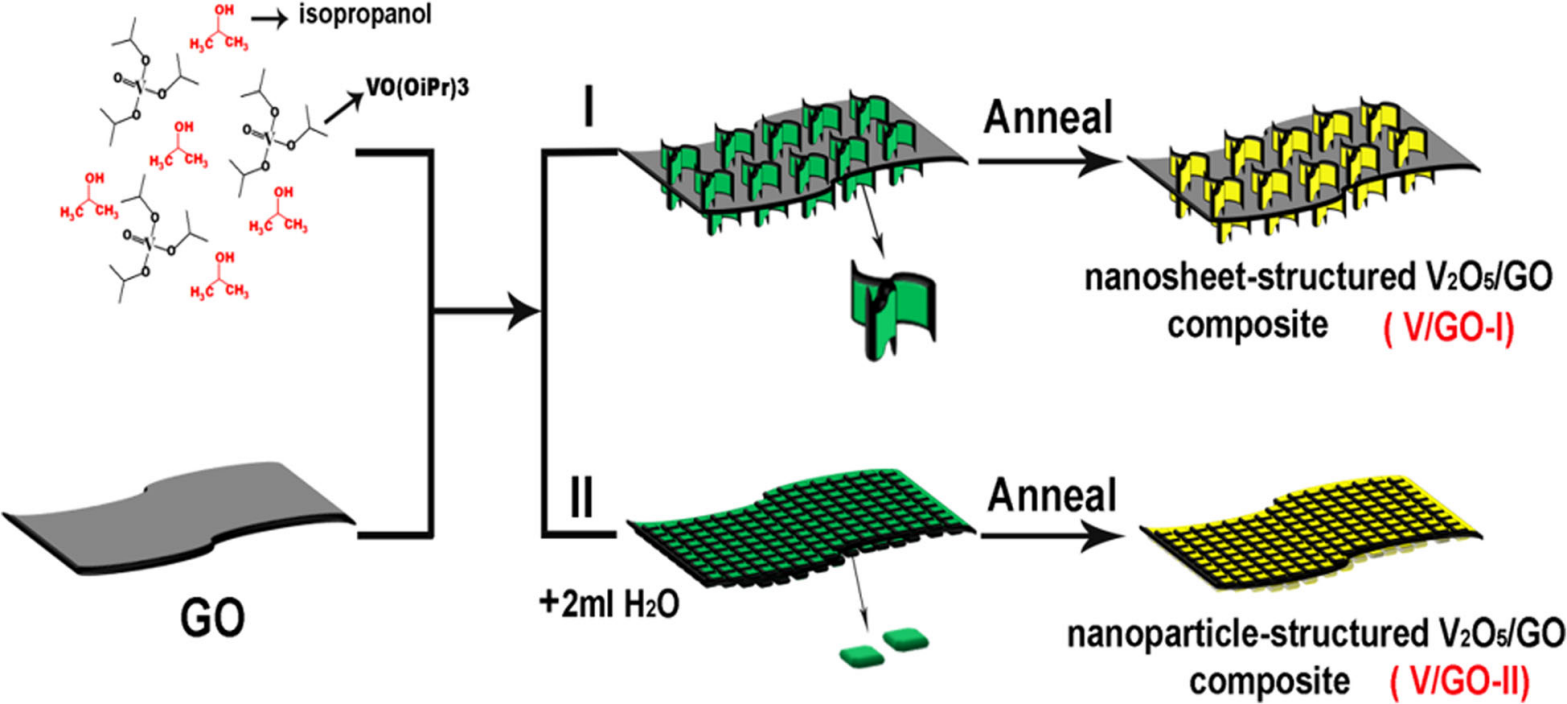
Schematic illustration of preparation of V_2_O_5_/graphene composites with nanosheet (I) and nanoparticle (II) assembled subunits. Process I and II indicate the solvothermal synthesis using different solvent compositions

Figure 1 illustrates the two different synthetic processes of V_2_O_5_/graphene nanocomposites with diverse self-assembled subunits. As indicated by process I, vanadium precursor nanosheets are grown on the graphene nanosheets first during the solvothermal process, in which vanadium triisopropoxy oxide (VO(O^i^Pr)3) was used as the vanadium resources. The prepared vanadium precursor and graphene composite can be converted into V_2_O_5_ and graphene composite with well-preserved structures. Process II indicates the preparation of nanoparticle-assembled vanadium precursor and graphene composite by adding extra 2 mL de-ion water in the solvothermal solution. Thereafter, the precursor composite can be converted into V_2_O_5_ nanoparticles and reduced graphene oxide nanocomposites (V/GO-II).

**Fig. 2:**
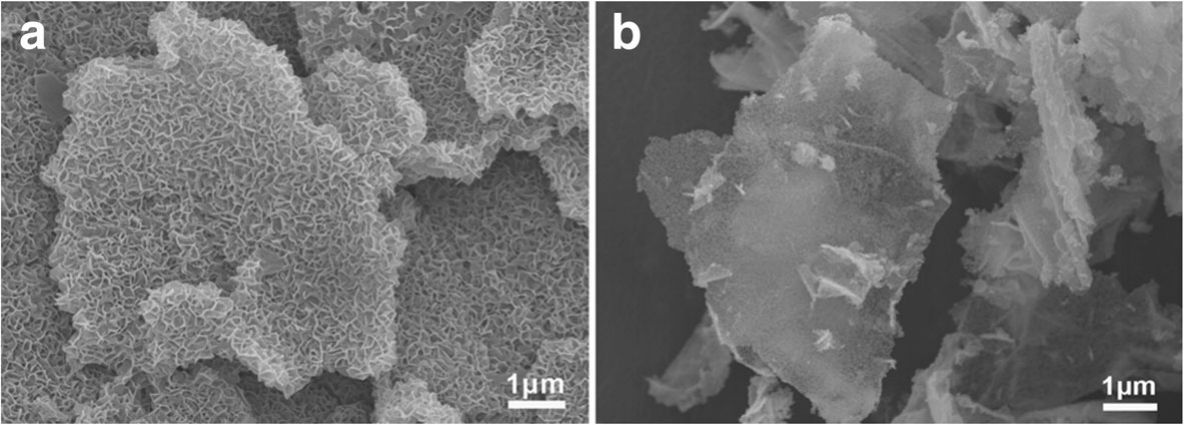
SEM images of the nanosheet-assembled vanadium precursor/GO composite (**a**) and nanoparticle-assembled vanadium precursor/GO composite (**b**)

Figure 2a shows the SEM image of the solvothermal products from process I, which indicates the homogenous distribution of vanadium precursor on graphene nanosheets. As we know, graphene oxides have large amount of functional groups on its surface, which is helpful for the growth of vanadium precursors. The vanadium precursors are composed of nanosheets and uniformly grew on the reduced graphene oxide substrates. The vanadium precursor nanosheets are about 1 μm in-plane and connect with neighboring nanosheets to form porous structure on the surface of graphene nanosheets. In the formation of the composite, graphene functions as a planar templates and the growth of nanosheets on its surface can greatly prevent their restacking and improve the electrolyte penetration. Figure 2b shows the FESEM image of the nanoparticle-assembled vanadium precursor and graphene composite. The vanadium precursor is of nanoparticle morphology and distributes homogeneously on the graphene nanosheets. Additional file 1: Figure S1 shows the XRD patterns of the solvothermally prepared two composites. The detection of broad XRD peaks around 30° can be attributed to the reduced graphene nanosheets. No other distinguishable XRD peaks are detected for the nanosheet-assembled vanadium precursor/GO composite, indicating the amorphous feature of the vanadium-precursor nanosheets. However, the XRD peak of the nanoparticle-assembled vanadium precursor/reduced graphene composite can be attributed to VO_2_ phase (space group: *P42/mnm* (136); *a* = 0.4556 nm, *b* = 0.4556 nm, *c* = 0.285 nm, JCPDS card no. 76-0677). The formation of VO_2_ phase can be attributed the reduction of V_2_O_5_·nH_2_O by alcohol during solvothermal process, in which V_2_O_5_·nH_2_O is prepared by the hydrolysis of vanadium triisopropoxy oxide with water. The result indicates the structures of the vanadium precursor can be adjusted by the addition of 2 mL de-ion water. The addition of water can speed the hydrolysis reaction between vanadium triisopropoxy oxide and water to form brown colored V_2_O_5_·nH_2_O, which may generate more nucleation on the graphene nanosheets, but sacrificing their parcel growth. However, due to the amorphous feature of the vanadium precursor nanosheets, it is difficult to give the exact reaction during the solvothermal process. The formation of the amorphous nanosheets may be attributed to the decomposition of vanadium triisopropoxy oxide under the condition of high temperature and high pressure during the solvothermal process. Much less nuclei seeds are formed on the reduced graphene oxide and grow continuously to form the nanosheet morphology without the addition of water.

**Fig. 3:**
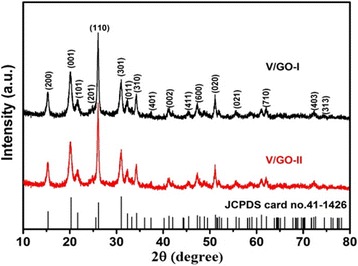
XRD patterns of the V/GO-I composite and the V/GO-II composite

**Fig. 4:**
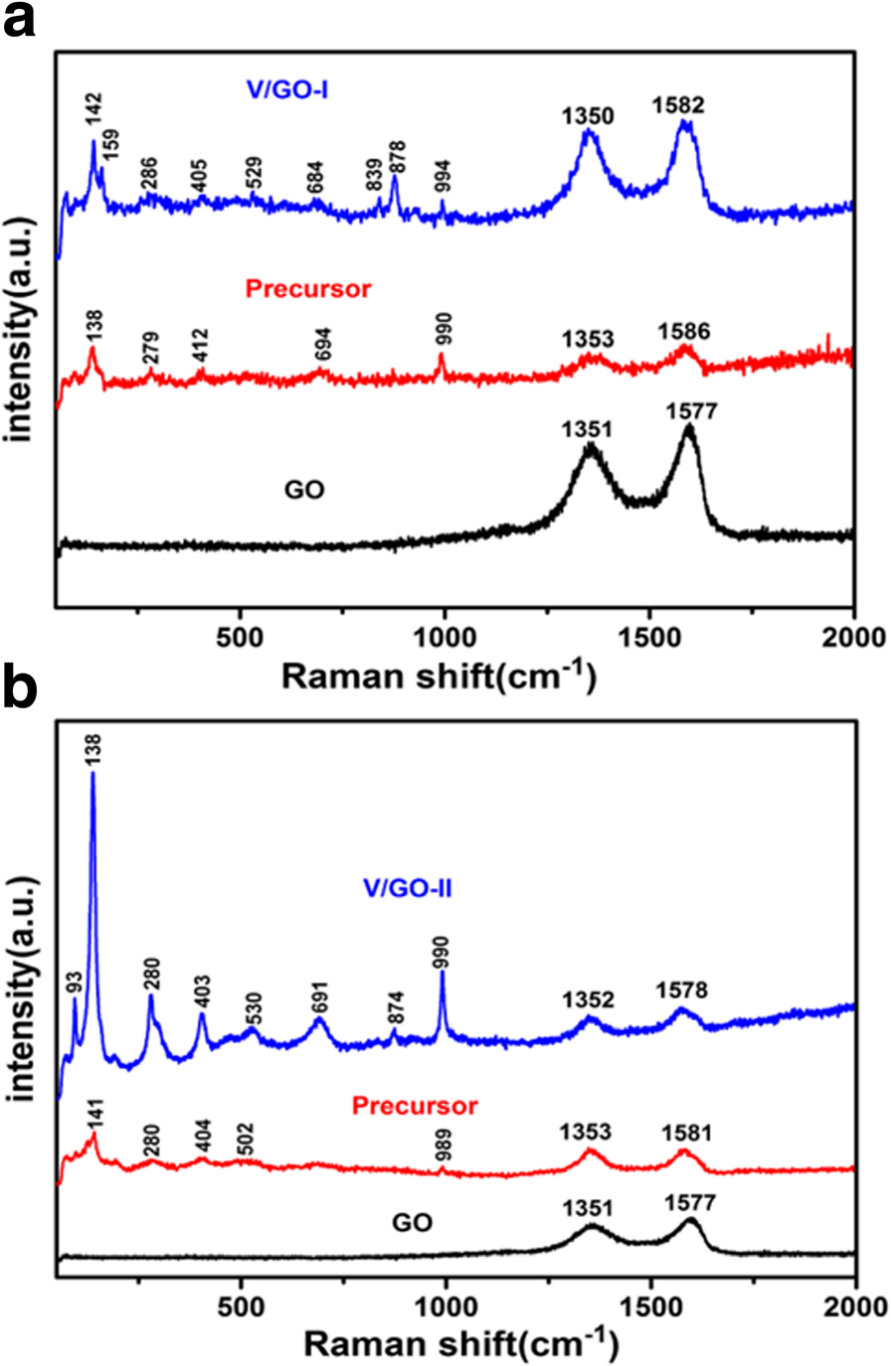
Raman spectrums of the V/GO-I composite (**a**) and the V/GO-II composite (**b**)

**Fig. 5:**
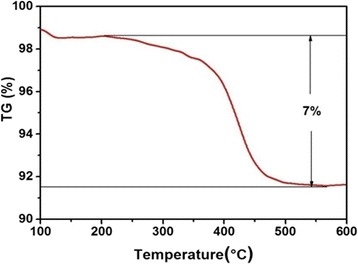
TG curve of the V/GO-I composite in air from 100 to 600 °C. The temperature ramp rate was 5 °C min^−1^

Figure 3 shows the powder X-ray diffraction (XRD) patterns of the V/GO-I composite and the V/GO-II composite. After calcination, the identified diffraction peaks can be indexed to the orthorhombic V_2_O_5_ phase (space group: *Pmmn* (no. 59); *a* = 1.1516 nm, *b* = 0.3565 nm, *c* = 0.4372 nm, JCPDS card no. 41-1426). The existence of reduced graphene oxides in the composite was confirmed by the Raman results, as shown in Fig. 4. The characteristic peaks at 1350 and 1580 cm^−1^ correspond to the typical G and D bands of graphene. Moreover, the intensity ratio between G and D bands become larger, indicating the partial reduction of graphene oxide. All other Raman peaks for the V/GO-I composite and the V/GO-II composite can be characterized on the basis of the vanadium oxide (Table 1) [28–30]. The weight percentage of graphene in the composite was determined by TG analysis and the result is shown in Fig. 5. The gentle weight loss before 200 ^o^Ccan be ascribed to the evaporation of physical adsorbed water, and the fast weight drop thereafter can be attributed to the burning out of reduced graphene oxides. Therefore, the content of reduced graphene oxide in the composite is approximately 7%.

**Table 1:** Raman peaks and their assignments of V_2_O_5_

Frequency (cm^−1^)	Assignment	Vibration modes
994	Stretching vibration of V=O	A_g_
684	Stretching vibration of V–O_1_	B_2g_ and B_3g_
529	Stretching vibration of V–O_2_	A_g_
405	Bending vibration of V–O_2_–V	A_g_
286	Bending vibration of O_3_–V–O_2_	B_2g_
138	Lattice vibration	B_3g_

**Fig. 6:**
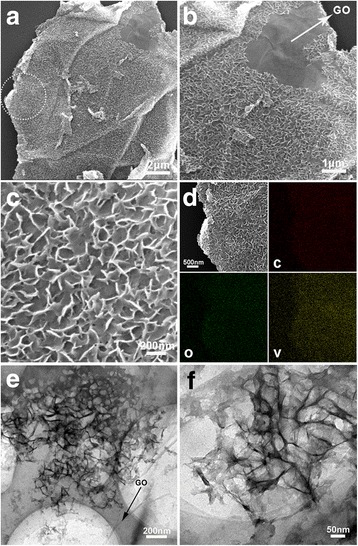
SEM (**a**, **b**, **c**), elemental mapping result (**d**), and TEM images (**e**, **f**) of the V/GO-I composite at different magnifications

Figure 6 shows the structural characterization of the V/GO-I composite, which is obtained by annealing the nanosheet-assembled vanadium precursor and reduced graphene oxides in air. According to FESEM image (Fig. 6a), the nanosheet morphology of the vanadium precursor can be well-preserved after conversion into V_2_O_5_. Moreover, the existence of graphene in the composite is also demonstrated by the broken part of the composite, as shown in Fig. 6b. The result is in good accordance with the Raman and TG analysis results. The high-magnification FESEM image (Fig. 6c) shows the V_2_O_5_ nanosheets are unparalleled to each other to form a porous three-dimensional structure, which may be of advantage of preventing the re-stack of graphene nanosheets and the electrolyte penetration. Figure 6d shows the elemental mapping results of the nanosheet-assembled V_2_O_5_ and reduced graphene oxide composites, which reveals the uniform distribution of C, V, and O elements in the composite. Figure 6e, f shows the TEM images of V_2_O_5_ nanosheets and graphene oxide composite, indicating the porous feature of the vanadium oxides and their high uniformity.

**Fig. 7:**
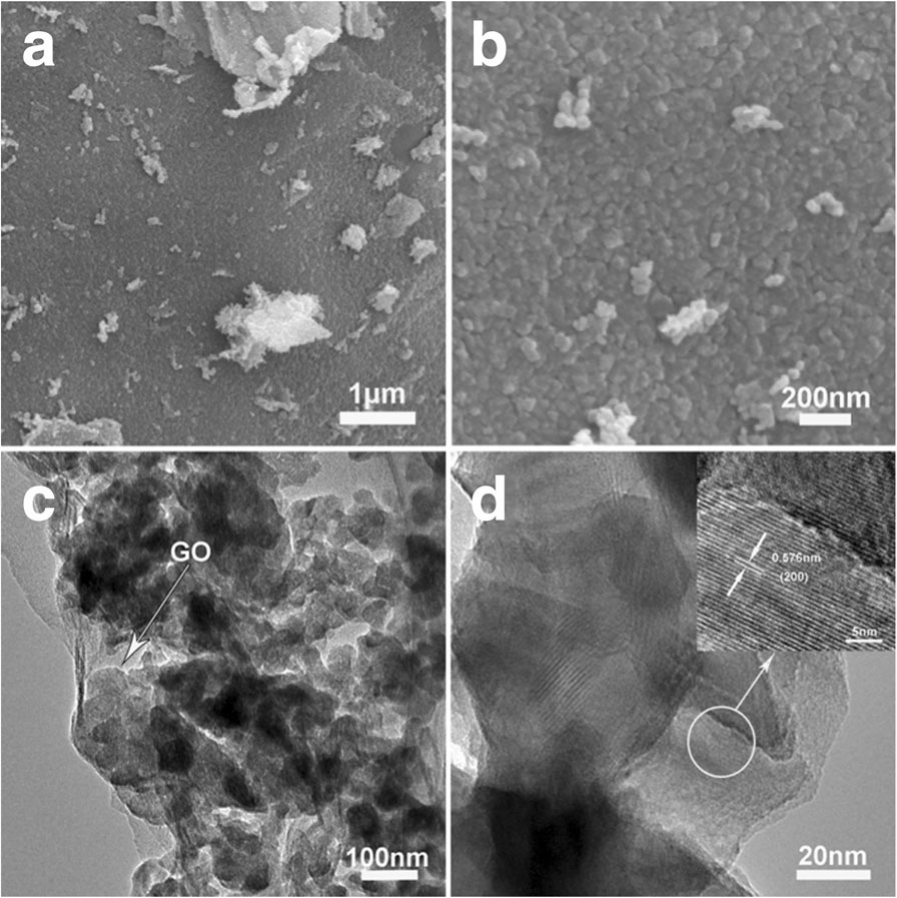
SEM (**a**, **b**) and TEM (**c**, **d**) images of the V/GO-II composite. The *inset* in (**d**) shows the HRTEM image of the V/GO-II composite

Figure 7 shows the FESEM and TEM images of the nanoparticle-assembled V_2_O_5_ and reduced graphene oxides. According to the FESEM images (Fig. 7a, b), the V_2_O_5_ nanoparticles in the composite is homogeneously distributed on reduced graphene oxide nanosheets, similar to the morphology of the nanoparticle-assembled vanadium precursor and reduced graphene oxide composite in Fig. 2b. The TEM images give more detailed information the structure. The nanoparticles are about 50–100 nm in-plane size. The HRTEM image (Fig. 7d) reveals the stacking feature of the nanoparticles and each nanoparticle is further composed of several closely stacked small nanosheets. Moreover, the lattice fringes are well observed with a distance of 0.576 nm, in good agreement with the planar distance of (200).

**Fig. 8:**
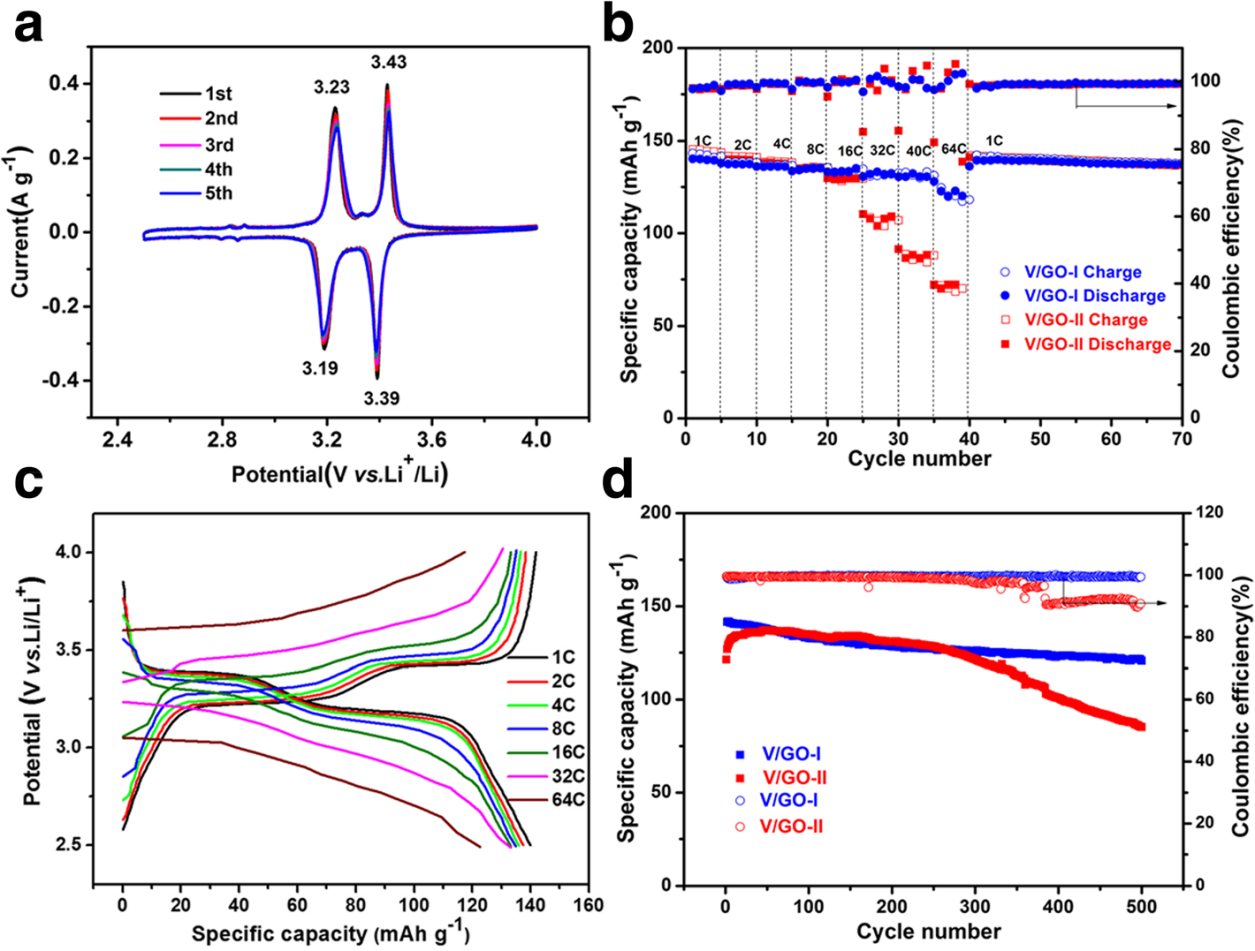
**a** CV curves of the V/GO-I composite at a scan rate of 0.1 mV s^−1^. **b** Rate capability of the V/GO-I composite and the V/GO-II composite at different current densities. **c** Discharge/charge voltage profiles of the V/GO-I composite at different current rates. **d** Cycling performance of the V/GO-I composite and the V/GO-II composite at a current rate of 2C

The V_2_O_5_ and reduced graphene oxide composites were assembled into coin cells to evaluate their electrochemical properties and the results are shown in Fig. 8. Figure 8a shows the first consecutive five CV curves for the V/GO-I composite at a scan rate of 0.1 mV s^−1^. Two pairs of redox peaks are clearly observed for each cycle, which indicates the multiple step phase transitions for V_2_O_5_ and reduced graphene oxide composite. The cathodic peaks at 3.39 and 3.19 V correspond to the phase transition from V_2_O_5_ to ε-Li_0.5_V_2_O_5_, then to δ-Li_1.0_V_2_O_5_, respectively. And the two corresponding anodic peaks at 3.23 and 3.43 V are related to the phase transformations from δ-Li_1.0_V_2_O_5_ to ε-Li_0.5_V_2_O_5_, and then to V_2_O_5_ in return [31–33]. No distinct peak intensity and potential position changes for the subsequent cycles are detected, suggesting the good reversibility of the electrode. Moreover, two weak redox peaks between 2.8 and 2.9 V can be detected, which may be attributed to the surface adoption or desorption of Li^+^ ions upon cycling. Comparing to the CV curves for V/GO-II composite (Additional file 1: Figure S2), the V/GO-I composite exhibit higher peak intensity and smaller polarization [34].

Figure 8b shows the rate performances of the composites. Both V/GO-I and V/GO-II composite electrodes exhibit good rate capability. The capacities vary slightly for the composites less than 8C. However, the rate performance of the V/GO-I composite becomes obvious at rates higher than 16C. A specific capacity of 133 mA h g^−1^ can be obtained for V/GO-I composite even at 16C, which is very close to the theoretical capacity of 147 mA h g^−1^ for one lithium-ion intercalation per formula. Moreover, almost no capacity fade is detected at 32C and 40C. Even at 64C, the V/GO-I composite can still deliver a specific discharge capacity of 122 mAh g^−1^. However, the capacity decreases much faster at high rates (>16C) for V/GO-II composite. The corresponding charge/discharge curves are shown in Additional file 1: Figure S3. The composite electrodes deliver specific discharge capacities of 108, 85, 74, and 50 mA h g^−1^ at 16C, 32C, 40C and 64C, respectively. When the discharge/charge rates were reset to 1C, high discharge capacity of 139 mAh g^−1^ can be recovered for both composites. The coulombic efficiency of V/GO-I composite is close to 100% in the whole discharge/charge process.

Figure 8c shows the charge/discharge profiles of the V/GO-I composite at various rates. The working potentials decrease gradually and the discharge/charge plateaus can still be observed even at higher rates. The voltage gap is much smaller and the capacity is larger than those for V/GO-II composite. The result indicates the lower polarization of the nanosheet-assembled V_2_O_5_/graphene composite, which may be attributed to the porous structures created by the nanosheets.

Figure 8d shows the long-term cycling performance for the two V_2_O_5_/graphene composites. The V/GO-I composite deliver an initial high specific capacity of 142 mAh g^−1^ at 2C and retain a capacity of 121 mA h g^−1^ after 500 cycles. The average capacity fading is only 0.03% per cycle. For comparison, the V/GO-II composite only retains a capacity of 80 mA h g^−1^ after 500 cycles at the same rate. Additional file 1: Figure S4 shows the charge/discharge profiles of the first, tenth, 100th, and 500th cycles at 2C for the two composites. As shown in Additional file 1: Figure S4a, The initial discharge and charge capacities are 142.1 and 141.6 mA h g^−1^, respectively, for the V/GO-I composite and the coulombic efficiency can be of 99.6%. After 500 cycles, the plateaus can still be easily detected. Additional file 1: Figure S4b shows the discharge/charge profiles for V/GO-II composite, which indicates the significant change of the plateaus for the 500th cycle. The result demonstrates the improved cycling stability of the V/GO-I composite.

**Fig. 9:**
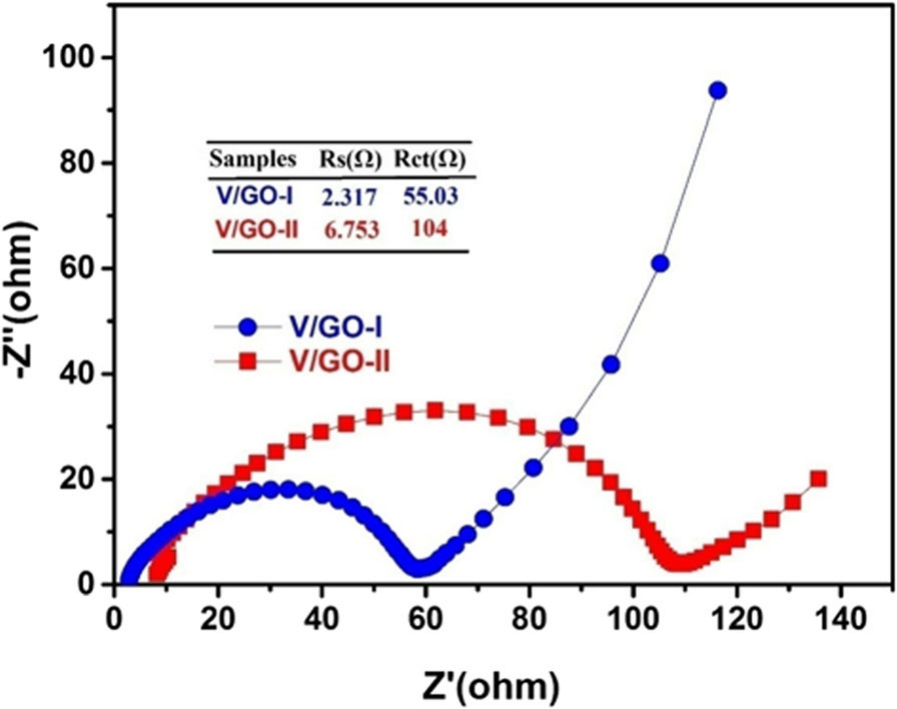
Nyquist plots and the equivalent circuit model as well as the simulated results for the V/GO-I composite and the V/GO-II composite

Figure 9 shows the electrochemical impedance spectroscopy (EIS) measurement results for both electrodes, which acquired from fresh cells. The semi-cycle for V/GO-I is much smaller than that for V/GO-II composite. According to the simulation result, the charge transfer resistance for V/GO-I is about 55.03 Ω, which is far less than 104 Ω for the V/GO-II composite. The result demonstrates the improved charge transfer kinetics for the nanosheet-assembled V_2_O_5_ and reduced graphene oxide composite. According to the electrochemical analysis results, the nanosheet-assembled V_2_O_5_/graphene composite exhibit better rate capability, cycling stability, and lower charge transfer resistance than the nanoparticle-assembled V_2_O_5_/graphene composite. The electrochemical performance of V/GO-I composite is also much better than many previously reported V_2_O_5_ electrodes, such as hollow structured V_2_O_5_ microspheres [35] and three-dimensional porous V_2_O_5_ [36]. Table 2 lists the rate performance of V_2_O_5_-based electrodes in this work and from many previous reported literatures. As shown in Table 2, the nanosheet-assembled V_2_O_5_/r-GO composite exhibit higher capacity and better rate capability than many other V_2_O_5_-based electrodes. The excellent rate performance and superior cyclic stability can be attributed to the synergistic effects between V_2_O_5_ and graphene substrates, which include the following aspects: (1) the growth of V_2_O_5_ nanosheets on reduced graphene oxide can ensure the good electronic conductivity of the electrode materials; (2) the porous space created by the interconnected large nanosheets can improve the accessibility of the electrolyte with the electrode materials; (3) the ultra-thin nanosheet thickness can greatly reduce the Li^+^ ions diffusion and electron transportation distances; and (4) the porous structure may better accommodate the volume changes upon cycling.

**Table 2:** Electrochemical properties of V_2_O_5_ and its hybrid composites

Electrode description	Voltage window (V)	Current density (mA g^−1^)	Specific capacity (mA h g^−1^)
V_2_O_5_ nanoparticles [37]	2.5–4	147 (1C)	138
		588 (4C)	116
		1176 (8C)	88
V_2_O_5_ nanosheets [32]	2.5–4	200 (1.4C)	138
		3200 (21.7C)	119
V_2_O_5_ nanospheres [12]	2.5–4	220 (1.5C)	136
		2205 (15C)	92
V_2_O_5_ nanobelts [31]	2.5–4	147 (1C)	139
		1176 (8C)	127
V_2_O_5_ microspheres [35]	2.5–4	147 (1C)	140
		2940 (20C)	120
V_2_O_5_ nanoribbon [38]	1.5–3.75	43.7 (0.1C)	290
		437 (1C)	190
		874 (2C)	120
V_2_O_5_@carbon composite [39]	2.5–4	147 (1C)	140
		2352 (16C)	125
		4704 (32C)	122
This work	2.5–4	147 (1C)	140.2
		2352 (16C)	133
		4704 (32C)	131

## Conclusions

In summary, nanosheet- or nanoparticle-assembled V_2_O_5_/reduced graphene oxide composites are synthesized by adjusting the solvothermal condition. The vanadium precursor can grow homogeneously on the reduced graphene oxides and the obtained composites can be converted into V_2_O_5_/reduced graphene oxide composites with good structural reservation. As cathode materials for lithium-ion batteries, the nanosheet-assembled V_2_O_5_/reduced graphene oxide nanocomposites (V/GO-I) exhibit better rate capability and cycling stability than the nanoparticle-assembled V_2_O_5_/graphene nanocomposites (V/GO-II). The superior electrochemical performance is attributed to the synergistic effects between V_2_O_5_ nanosheets and reduced graphene oxides.

## Additional file

Additional file 1: XRD patterns of vanadium precursors, CV curves, charge/discharge profiles of the V@GO-II composite. Discharge/charge voltage profiles of the V@GO-I composite. Raman peaks and their assignments of V_2_O_5_. Figure S1. XRD patterns of the nanosheet-assembled vanadium precursor/GO composite (blue line) and the nanoparticle-assembled vanadium precursor/GO composite (red line). Figure S2. CV curves of the V/GO-II composite at a scan rate of 0.1 mV s^−1^. Figure S3. Charge/discharge profiles of the V@GO-II composite at different densities. Figure S4. Discharge/charge voltage profiles of the V@GO-I composite (a) and the V@GO-I composite (b) at a current rate of 2C. (DOC 4760 kb)

## Abbreviations

CV: Cyclic voltammetry
FESEM: Field emission scanning electron microscopy
GO: Graphene oxide
TEM: Transmission electron microscopy
V/GO-I: Nanosheet-structured V_2_O_5_/reduced graphene oxide composite I
V/GO-II: Nanoparticle-structured V_2_O_5_/reduced graphene oxide composite II
XRD: X-ray diffraction
