# Supplementary material for: Controllable Preparation of V2O5/Graphene Nanocomposites as Cathode Materials for Lithium-Ion Batteries
Source: Nanoscale Res Lett. 2016 Dec 12;11:549. doi: 10.1186/s11671-016-1764-3 (PMC5153389; doi:10.1186/s11671-016-1764-3)
Supplement: Additional file 1: — XRD patterns of vanadium precursors, CV curves, charge/discharge profiles of the V@GO-II composite. Discharge/charge voltage profiles of the V@GO-I composite. Raman peaks and their assignments of V2O5. Figure S1. XRD patterns of the nanosheet-assembled vanadium precursor/GO composite (blue line) and the nanoparticle-assembled vanadium precursor/GO composite (red line). Figure S2. CV curves of the V/GO-II composite at a scan rate of 0.1 mV s−1. Figure S3. Charge/discharge profiles of the V@GO-II composite at different densities. Figure S4. Discharge/charge voltage profiles of the V@GO-I composite (a) and the V@GO-I composite (b) at a current rate of 2C. (DOC 4760 kb) [file 11671_2016_1764_MOESM1_ESM.doc]

**Supplementary Information**

**Controllable preparation of V_2_O_5_/Graphene nanocomposites as cathode materials for lithium ion batteries**

Yanglin Liu, ^†, ‡^YapingWang, ^†^,* YifangZhang, ^†^ShuquanLiang, ^†^,Anqiang Pan, ^†^,*

^†^ School of Materials Science & Engineering, Central South University, Changsha, 410083, Hunan, China

^‡^Changsha Environmental Protection Vocational College, Changsha 410004, Hunan, China

**AUTHOR INFORMATION**

**Corresponding Authors**

*E-mail: [pananqiang@csu.edu.cn](mailto:pananqiang@csu.edu.cn) (A. Pan);

*E-mail: [yapingwang@csu.edu.cn (Y.P](mailto:yapingwang@csu.edu.cn (Y.P). Wang);

**
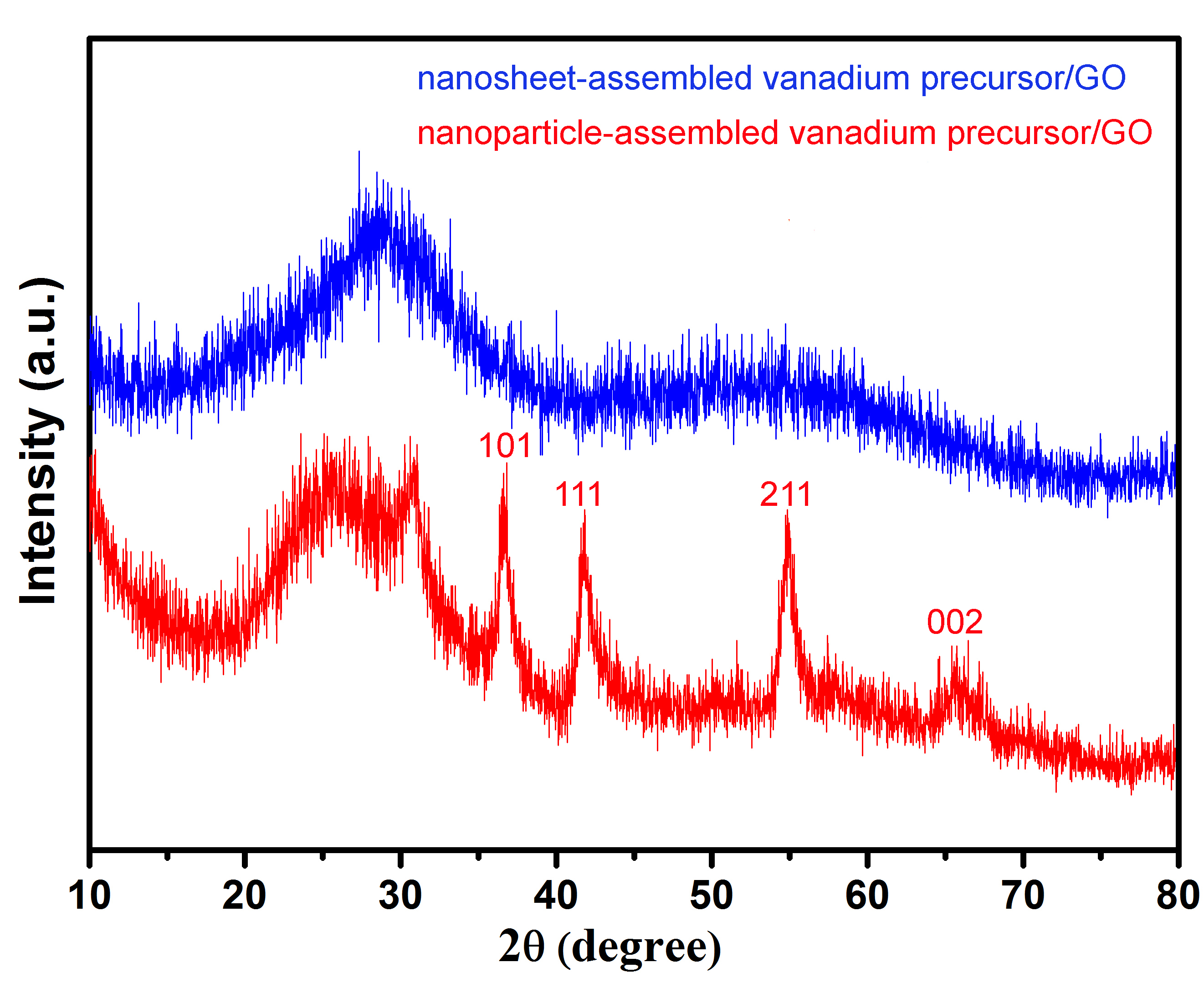
**

**FigureS1** XRD patterns of the nanosheet-assembled vanadium precursor/GO composite (blue line) and the nanoparticle-assembled vanadium precursor/GO composite (red line).


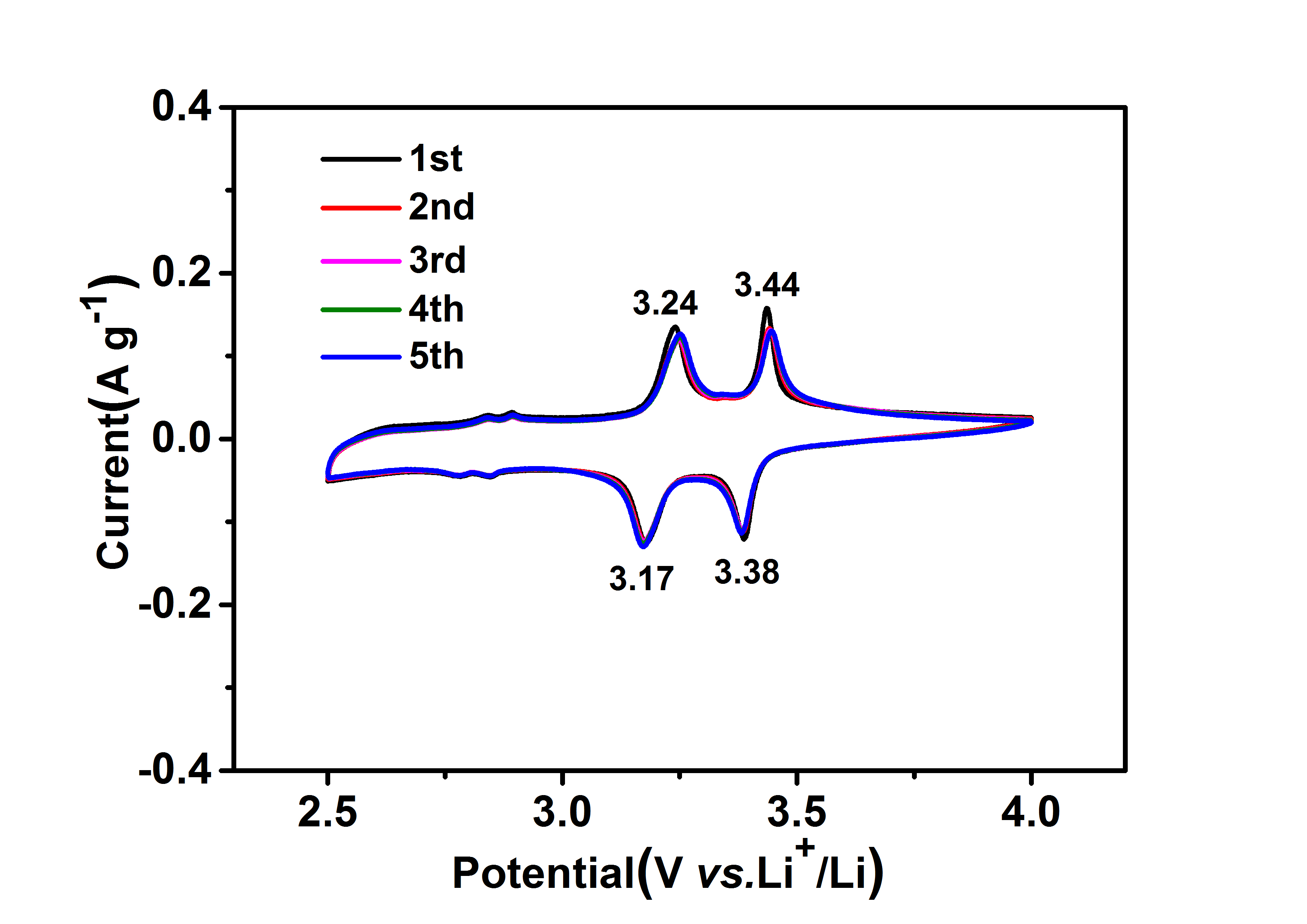


**FigureS2** CV curves of the V/GO-II composite at a scan rate of 0.1mV s^-1^.


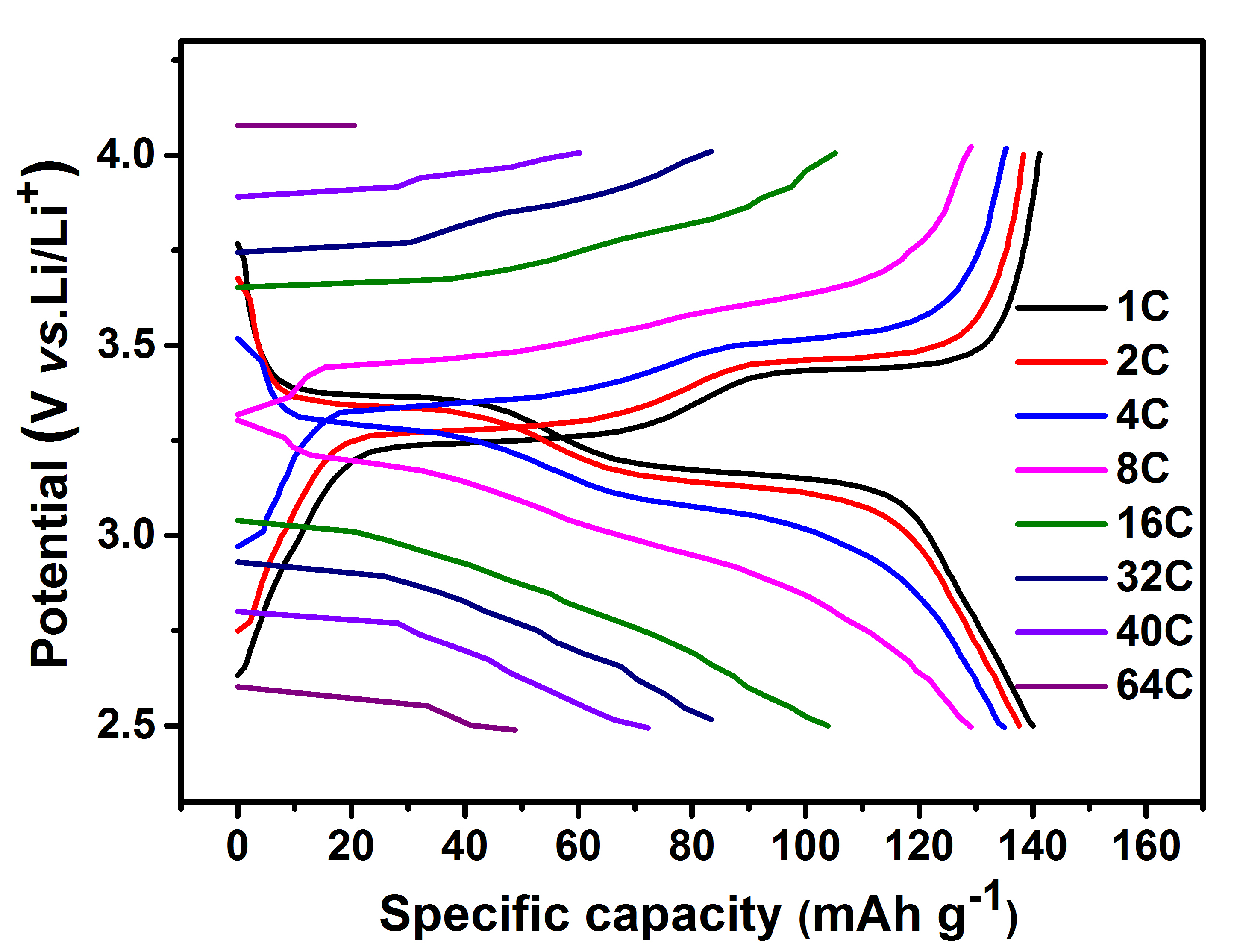


**FigureS3** Charge/discharge profiles of the V@GO-II composite at different densities.


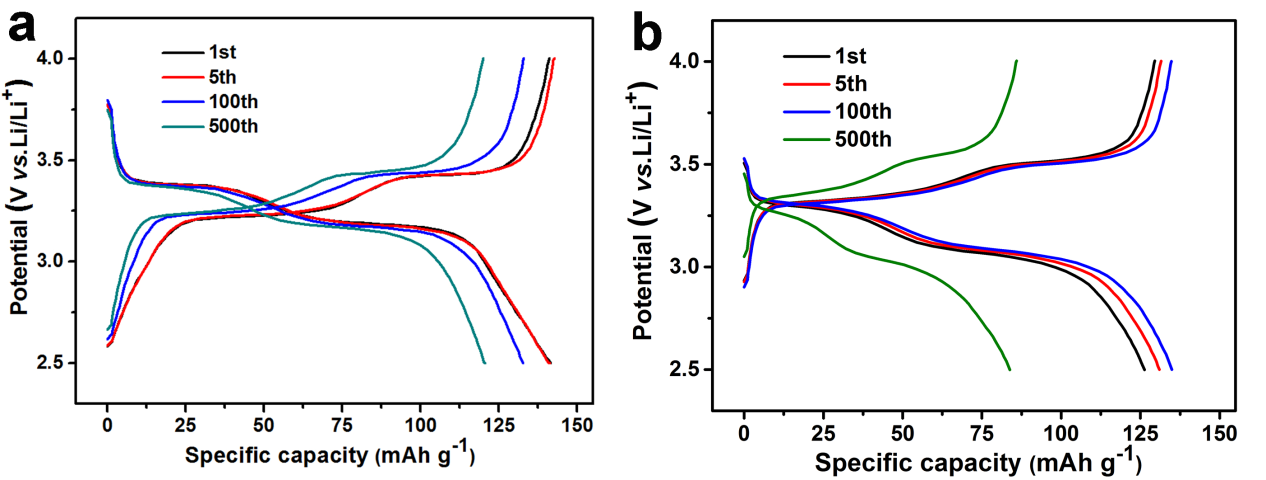


**FigureS4** Discharge/charge voltage profiles of the V@GO-I composite (a) and the V@GO-I composite (b) at a current rate of 2C.
